# Anticipatory Caregiving Scale: Development and Preliminary Validation

**DOI:** 10.1093/geroni/igab046.2976

**Published:** 2021-12-17

**Authors:** Kendall Weber, Sara Qualls

**Affiliations:** 1 University of Colorado at Colorado Springs, University of Colorado at Colorado Springs, Colorado, United States; 2 University of Colorado Colorado Springs, University of Colorado at Colorado Springs, Colorado, United States

## Abstract

Background: As public awareness of family caregiving has grown, adults likely anticipate the role they may play as a caregiver for an aging loved one. Although anticipatory planning for caregiving has been studied, no measure of multiple dimensions of the anticipated caregiving experience exists. The purpose of the present study is to develop and validate the Anticipatory Caregiving Scale (ACS), an assessment of adult children’s attitudes toward and level of expectation surrounding their potential role as an informal caregiver to a parent or parent-in-law. Method: The ACS consists of six subscales that assess multiple factors that may influence willingness to or expectations about taking on a caregiving role: affect surrounding future caregiving, anticipated lifestyle interference, self-efficacy surrounding future caregiving, anticipated caregiving resources, endorsement of norms of family care which influence anticipated caregiving, and the relationship quality, current and anticipated, with the potential care recipient. A sample of 540 adults aged 18 and over recruited online completed the ACS, along with scales to assess convergent, discriminant, and concurrent validity. Results: Overall, the ACS and its subscales demonstrated good reliability and validity, established through internal consistency, and convergent, discriminant, and concurrent validity. Discussion: The ACS fills a gap in the current literature by providing a comprehensive, multidimensional assessment examining expectations about one’s potential caregiving experience, specific to the adult child-parent dyad. Future studies should examine the psychometric properties of the ACS in a more diverse population of adults across different settings and assess the temporal stability and criterion validity of the scale.

